# Molecular characterization and zoonotic potential of *Entamoeba* spp., *Enterocytozoon bieneusi* and *Blastocystis* from captive wild animals in northwest China

**DOI:** 10.1186/s12917-024-04172-y

**Published:** 2024-07-10

**Authors:** Yuexin Wang, Yuechen Zeng, Yaoli Wu, Furui Lu, Xiaopeng Hou, Junfeng Shao, Tengteng Zhang, Chen Shao

**Affiliations:** 1 Laboratory of Biodiversity and Evolution of Protozoa, College of Life Sciences, Shaanxi Normal University, Xi’an, Shaanxi China; 2 Xi’an Dahanshanglinyuan Industrial Co., Ltd, Xi’an, Shaanxi China

**Keywords:** *Enterocytozoon bieneusi*, *Entamoeba* spp., *Blastocystis*, Wildlife, Prevalence, Phylogeny, Zoonotic potential

## Abstract

**Background:**

Parasites *Entamoeba* spp., *Enterocytozoon bieneusi* and *Blastocystis* are prevalent pathogens causing gastrointestinal illnesses in animals and humans. Consequently, researches on their occurrence, distribution and hosts are crucial for the well-being of both animals and humans. Due to the confined spaces and frequent interaction between animals and humans, animal sanctuaries have emerged as potential reservoirs for these parasites. In this study, the wildlife sanctuary near the Huang Gorge of the Qinling Mountains in northwest China is chosen as an ideal site for parasite distribution research, considering its expansive stocking area and high biodiversity.

**Results:**

We collected 191 fecal specimens from 37 distinct wildlife species and extracted genomic DNA. We identified these three parasites by amplifying specific gene regions and analyzed their characteristics and evolutionary relationships. All the parasites exhibited a high overall infection rate, reaching 90.05%. Among them, seven *Entamoeba* species were identified, accounting for a prevalence of 54.97%, with the highest infection observed in *Entamoeba bovis*. In total, 11 *Enterocytozoon bieneusi* genotypes were discovered, representing a prevalence of 35.08%, including three genotypes of human-pathogenic Group 1 and two novel genotypes (SXWZ and SXLG). Additionally, 13 *Blastocystis* subtypes were detected, showing a prevalence of 74.87% and encompassing eight zoonotic subtypes. All of the above suggests significant possibilities of parasite transmission between animals and humans.

**Conclusions:**

This study investigated the occurrence and prevalence of three intestinal parasites, enhancing our understanding of their genetic diversity and host ranges in northwest China. Furthermore, the distribution of these parasites implies significant potential of zoonotic transmission, underscoring the imperative for ongoing surveillance and implementation of control measures. These efforts are essential to mitigate the risk of zoonotic disease outbreaks originating from wildlife sanctuary.

**Supplementary Information:**

The online version contains supplementary material available at 10.1186/s12917-024-04172-y.

## Background

Protozoa, the unicellular eukaryotes with global distribution and high biodiversity, encompass numerous parasitic species [1–4]. Among them, *Entamoeba* spp., *Enterocytozoon bieneusi*, and *Blastocystis* are three predominant protozoan parasites that infect various animals and humans, leading to diarrhea and gastrointestinal illnesses [5–7].

The parasite *Entamoeba* spp. has a wide range of hosts, including humans and animals [8–10]. Among *Entamoeba* species, *Entamoeba histolytica* is classified as a category B priority biodefense pathogen, and could cause amebiasis with diarrhea and liver abscesses as main symptom, leading to approximately 100,000 human deaths per year globally [6, 11–14]. Furthermore, some *Entamoeba* species have been identified as potential animal reservoirs. For instance, *Entamoeba chattoni* and *Entamoeba polecki* are prevalent in non-human primates, *Entamoeba moshkovskii* and *Entamoeba nuttalli* are found to be pathogenic in mice, and *Entamoeba suis* is commonly found in swine [15–19]. *Enterocytozoon bieneusi*, one of the most commonly diagnosed pathogens, possesses over 500 genotypes grouped into 11 major groups, including the zoonotic Group 1 with some genotypes (e.g., WL12 and NIA1) posing a threat to public health [20–24]. Besides, the *Enterocytozoon bieneusi* could be transmitted through water and possess a wide host range of mammalian and avian. It is also responsible for over 90% of documented cases of human microsporidiosis with varying clinical symptoms, typically diarrhea and wasting [22, 25]. *Blastocystis* can parasitize the colon and caecum of reptiles, birds, and mammals [26]. Furthermore, it exhibits global infections in humans, with infection rates exceeding 45% in certain countries [27]. Notably, *Blastocystis* is well-known for its morphological polymorphism and genetic diversity, with at least 35 proposed subtypes (STs), including widely acknowledged and accepted subtypes ST1-ST17, ST21, ST23-ST32 and ST42-ST44 [28–30]. Among these subtypes, ST1-ST9 are known to infect humans, and the remaining subtypes are predominantly found in animals [26].

Transmission of these parasites primarily occurs through the fecal-oral cycle and intimate contact, emphasizing the potential of zoonotic transmission through contacting with host animals [5, 7, 21, 31, 32]. Thus, epidemiological research on these three pathogens and their potential hosts is crucial for the health of both animals and humans. Due to the higher prevalence and transmission of parasites in captive wildlife protection areas, attributed to limited living space and extensive contact among animals and humans, these areas have become important investigation sites [33–35]. In recent years, compared to other regions in China [36–40], the northwest region has undergone fewer comprehensive investigations on intestinal parasites in wild animals. In this study, the wildlife sanctuary near the Huang Gorge of the Qinling Mountains in northwest China possesses rich biodiversity and expansive terrain, and also presents a more primitive and realistic ecotope. These factors make it an ideal sampling site to explore the transmission patterns of intestinal parasites.

The objectives of this research are to investigate the occurrence and distribution patterns of three prevalent parasites—*Enterocytozoon bieneusi*, *Entamoeba*, and *Blastocystis*—in wildlife sanctuary in Shaanxi Province, northwest China. Our study also helps to assess the possibilities of parasite transmission between humans and animals, and provide insights into the protection management of wildlife, as well as the prevention and control of zoonotic diseases.

## Results

### Occurrence of *Entamoeba* spp., *Enterocytozoon bieneusi*, and *Blastocystis*

Among the 191 fecal samples, 172 samples (90.05%, 95% CI: 85.8–94.3) were positive for the three pathogens (Table 1). The prevalence rates for *Entamoeba* spp., *Enterocytozoon bieneusi*, and *Blastocystis* were 54.97% (105/191, 95% CI: 47.9–62.1), 35.08% (67/191, 95% CI: 28.2–41.9), and 74.87% (143/191, 95% CI: 68.7–81.1), respectively (Table 1). Additionally, co-infection results indicated that 19 samples (9.95%, 95% CI: 68.7–81.1) were concurrently infected by three parasites, and 104 specimens showed co-infection by two species of parasites. Among these, 31 samples (16.23%, 95% CI: 11.0–21.5) and 73 samples (38.33%, 95% CI: 31.5–45.2) were simultaneously infected by *Blastocystis* + *Enterocytozoon bieneusi* and *Entamoeba* spp. + *Blastocystis*, respectively, while no specimens co-infected by *Entamoeba* spp. and *Enterocytozoon bieneusi* were detected (Table 1).

**Table 1:** Occurrence of *Entamoeba* spp., *Enterocytozoon bieneusi*, and *Blastocysti* in wild animals of the sanctuary in this study

Species	No. Positive/Samples	Prevalence	95% CI	Scientific name (No. Positive)
***Entamoeba *** **spp.**	105/191	54.97%	47.9–62.1	*Aepyceros melampus* (10); *Tragelaphus oryx* (10); *Cervus nippon* (6); *Chinese pony* (4); *Equus quagga* (4); *Budorcas taxicolor* (4); *Bos mutus* (10); *Struthio camelus* (4); *Ovis ammon* (8); *Cervus canadensis* (3); *Camelus bactrianus* (4); *Connochaetes taurinus* (15); *Sheland pony* (2); *Macaca arctoides* (9); *Lemur catta* (1); *Lama glama* (5); *Panthera tigris altaica* (1); *Panthera tigris tigris* (1); *Giraffa camelopardalis* (3); *Elephas maximus* (1)
***Enterocytozoon bieneusi***	67/191	35.08%	28.2–41.9	*Aepyceros melampus* (2); *Tragelaphus oryx* (1); *Cervus nippon* (3); *Equus quagga* (1); *Ovis ammon* (4); *Cervus canadensis* (1); *Connochaetes taurinus* (3); *Macaca arctoides* (3); *Lemur catta* (2); *Sapajus flavius* (3); *Ursus thibetanus* (14); *Lama glama* (1); *Panthera leo* (2); *Panthera pardus fusca* (1); *Hystrix brachyura hodgsoni* (1); *Ailurus fulgens* (1); *Panthera leo × Tigris* (1); *Nyctereutes procyonoides* (1); *Panthera tigris altaica* (13); *Panthera tigris tigris* (3); *Giraffa camelopardalis* (3); *Elephas maximus* (1); *Ceratotherium simum* (2)
***Blastocystis***	143/191	74.87%	68.7–81.1	*Aepyceros melampus* (13); *Tragelaphus oryx* (10); *Cervus nippon* (8); *Equus quagga* (1); *Budorcas taxicolor* (4); *Bos mutus* (10); *Ovis ammon* (8); *Cervus canadensis* (3); *Camelus bactrianus* (4); *Connochaetes taurinus* (15); *Dendrolagus mbaiso* (2); *Sheland pony* (1); *Rhinopithecus roxellanae* (3); *Macaca arctoides* (10); *Cercopithecus neglectus* (1); *Lemur catta* (2); *Sapajus flavius* (4); *Ursus thibetanus* (15); *Procyon lotor* (1); *Nyctereutes procyonoides* (1); *Canis lupus* (1); *Panthera tigris altaica* (5); *Lama glama* (6); *Giraffa camelopardalis* (3); *Panthera tigris tigris* (6); *Elephas maximus* (1); *Ceratotherium simum* (5)
***Entamoeba *** **spp. and ** ***Enterocytozoon bieneusi *** **(only)**	0/191	-	-	-
***Blastocystis *** **and ** ***Enterocytozoon bieneusi *** **(only)**	31/191	16.23%	11.0–21.5	*Aepyceros melampus* (1); *Cervus nippon* (1); *Lemur catta* (1); *Sapajus flavius* (3); *Ursus thibetanus* (14); *Nyctereutes procyonoides* (1); *Panthera tigris altaica* (4); *Lama glama* (1); *Panthera tigris tigris* (3); *Ceratotherium simum* (2)
***Entamoeba *** **spp. and ** ***Blastocystis *** **(only)**	73/191	38.33%	31.3–45.2	*Aepyceros melampus* (9); *Tragelaphus oryx* (9); *Cervus nippon* (4); *Equus quagga* (1); *Budorcas taxicolor* (4); *Bos mutus* (10); *Ovis ammon* (4); *Cervus canadensis* (2); *Camelus bactrianus* (4); *Connochaetes taurinus* (12); *Sheland pony* (1); *Macaca arctoides* (6); *Lemur catta* (5); *Panthera tigris altaica* (1); *Panthera tigris tigris* (1)
***Entamoeba *spp.**, ***Blastocystis *and *Enterocytozoon bieneusi***	19/191	9.95%	5.7–14.2	*Aepyceros melampus* (1); *Cervus nippon* (1); *Tragelaphus oryx* (1); *Ovis ammon* (4); *Cervus canadensis* (1); *Connochaetes taurinus* (3); *Macaca arctoides* (3); *Lemur catta* (1); *Giraffa camelopardalis* (3); *Elephas maximus* (1)
**total**	172/191		85.8–94.3	-

Note: Negative results are denoted by hyphens (“-”)

### Distribution of *Entamoeba* species in captive wild animals

Through amplification and sequencing of the SSU rRNA gene locus of *Entamoeba*, a total of 105 positive samples were successfully identified, corresponding to seven *Entamoeba* species: *Entamoeba bovis* (number (n) = 74), *Entamoeba* sp. RL9 (n = 11), *Entamoeba hartmanni* (n = 2), *Entamoeba chattoni* (n = 5), *Entamoeba polecki* (n = 3), *Entamoeba* sp. MG107/BEL (n = 6), and *Entamoeba suis* (n = 4) (Table 2). Among them, *Entamoeba bovis* was predominantly detected in the feces of herbivorous animals, such as *Aepyceros melampus* and *Tragelaphus oryx*, with only a small proportion found in omnivorous animals like tigers (*Panthera tigris altaica* and *Panthera tigris tigris*) (Table 2). *Entamoeba suis* was also identified in both herbivorous animals (*Lemur catta* and *Struthio camelus*) and omnivorous animals (*Macaca arctoides*). Furthermore, *Entamoeba hartmanni* and *Entamoeba chattoni* were exclusively found in monkeys (*Macaca arctoides*), while the remaining three *Entamoeba* taxa were solely present in herbivorous animals (Table 2).

**Table 2:** Distribution of *Entamoeba* spp., *Enterocytozoon bieneusi*, and *Blastocystis* among animals of the sanctuary in this study

	Species/Subtypes/Genotypes (No. Positive)	Scientific name (No. Positive)
***Entamoeba *** **spp.**	*Entamoeba bovis* (74)	*Aepyceros melampus* (10); *Tragelaphus oryx* (10); *Cervus nippon* (6); *Budorcas taxicolor* (4); *Bos mutus* (10); *Ovis ammon* (5); *Cervus canadensis* (3); *Camelus bactrianus* (4); *Connochaetes taurinus* (15); *Lama glama* (5); *Panthera tigris altaica* (1); *Panthera tigris tigris* (1)
	*Entamoeba* sp. RL9 (11)	*Chinese pony* (4); *Equus quagga* (4); *Sheland pony* (2); *Elephas maximus* (1)
	*Entamoeba hartmanni* (2)	*Macaca arctoides* (2)
	*Entamoeba chattoni* (5)	*Macaca arctoides* (5)
	*Entamoeba polecki* (3)	*Struthio camelus* (3)
	*Entamoeba* sp. MG107/BEL (6)	*Ovis ammon* (3); *Giraffa camelopardalis* (3)
	*Entamoeba suis* (4)	*Lemur catta* (1); *Struthio camelus* (1); *Macaca arctoides* (2)
**Total**	105	
***Enterocytozoon bieneusi***	SXWZ (16)	*Aepyceros melampus* (2); *Tragelaphus oryx* (1); *Connochaetes taurinus* (3); *Panthera leo* (1); *Panthera pardus fusca* (1); *Hystrix brachyura hodgsoni* (1); *Ailurus fulgens* (1); *Panthera leo × Tigris* (1); *Panthera tigris altaica* (5);
	BEB6 (4)	*Cervus nippon* (3); *Cervus canadensis* (1)
	JLD-VIII (13)	*Equus quagga* (1); *Ovis ammon* (4); *Macaca arctoides* (1); *Ursus thibetanus* (1); *Nyctereutes procyonoides* (1); *Panthera leo* (1); *Panthera tigris altaica* (2); *Panthera tigris tigris* (1); *Ceratotherium simum* (1)
	D (5)	*Macaca arctoides* (2); *Panthera tigris altaica* (1); *Panthera tigris tigris* (2)
	Type IV (2)	*Lemur catta* (2)
	CM4 (8)	*Sapajus flavius (3)*; *Panthera tigris altaica* (1); *Ursus thibetanus* (2); *Elephas maximus* (1); *Ceratotherium simum* (1)
	CHG21 (4)	*Panthera tigris altaica* (4)
	SXLG (1)	*Lama glama* (1)
	MJ5 (9)	*Giraffa camelopardalis* (3); *Ursus thibetanus* (6)
	CHB1 (3)	*Ursus thibetanus* (3)
	SC02 (2)	*Ursus thibetanus* (2)
**Total**	67	
***Blastocystis***	ST1 (3)	*Cercopithecus neglectus* (1); *Sapajus flavius* (1); *Panthera tigris altaica* (1)
	ST2 (2)	*Macaca arctoides* (2)
	ST3 (35)	*Macaca arctoides* (8); *Lemur catta* (2); *Sapajus flavius* (3); *Ursus thibetanus* (15); *Procyon lotor* (1); *Panthera tigris altaica* (2); *Panthera tigris tigris* (3); *Ceratotherium simum* (1)
	ST4 (1)	*Elephas maximus* (1)
	ST5 (4)	*Dendrolagus mbaiso* (2); *Sheland pony* (1); *Lama glama* (1)
	ST10 (49)	*Aepyceros melampus* (4); *Tragelaphus oryx* (8); *Cervus nippon* (8); *Equus quagga* (1); *Budorcas taxicolor* (4); *Bos mutus* (6); *Ovis ammon* (6); *Connochaetes taurinus* (6); *Nyctereutes procyonoides* (1); *Canis lupus* (1); *Panthera tigris altaica* (2); *Panthera tigris tigris* (1); *Ceratotherium simum* (1)
	ST12 (3)	*Giraffa camelopardalis* (3)
	ST13 (3)	*Rhinopithecus roxellanae* (3)
	ST14 (19)	*Aepyceros melampus* (1); *Tragelaphus oryx* (2); *Bos mutus* (1); *Ovis ammon* (1); *Ceratotherium simum* (2); *Lama glama* (5); *Cervus canadensis* (3); *Camelus bactrianus* (4)
	ST21 (7)	*Aepyceros melampus* (4); *Panthera tigris tigris* (2); *Ceratotherium simum* (1)
	ST23 (1)	*Ovis ammon* (1)
	ST25 (13)	*Aepyceros melampus* (4); *Connochaetes taurinus* (9);
	ST26 (3)	*Bos mutus* (3)
**Total**	143	

### Detection of *Enterocytozoon bieneusi* genotypes

Totally, 11 different genotypes of *Enterocytozoon bieneusi* were identified in this study, in which 9 genotypes were previously reported and well-known, including genotypes BEB6 (n = 4), JLD-VIII (n = 13), D (n = 5), Type IV (n = 2), CM4 (n = 8), CHG21 (n = 4), MJ5 (n = 9), CHB1 (n = 3), and SC02 (n = 2) (Table 2). Notably, two novel genotypes were discovered through comparison with reference sequences (MT895456 of genotype SH_deer1 and KX276713 of genotype horse2). One was named genotype SXWZ, displaying four and nine single nucleotide polymorphisms (SNPs) in the ITS region when compared to the above two reference sequences (Additional file 2: Fig. S4). It was widely distributed among herbivores and omnivores, such as antelopes, wildebeests, lions, and tigers (Table 2). The other one was named genotype SXLG, exclusively detected in an alpaca specimen, exhibiting three SNPs compared to reference genotypes CM4 (KF543866) and CHG21 (KP262376) (Additional file 2: Fig. S5). Moreover, among all *Enterocytozoon bieneusi* genotypes identified in this study, genotype SXWZ was the most prevalent one (23.88%, 16 out of 67), followed by genotype JLD-VIII (19.40%, 13 out of 67) (Table 2).

### Prevalence of *Blastocystis* subtypes

The sequence comparison of the SSU rRNA gene revealed the presence of 13 distinct *Blastocystis* subtypes, namely ST1 (n = 3), ST2 (n = 2), ST3 (n = 35), ST4 (n = 1), ST5 (n = 4), ST10 (n = 49), ST12 (n = 3), ST13 (n = 3), ST14 (n = 19), ST21 (n = 7), ST23 (n = 1), ST25 (n = 13), and ST26 (n = 3) (Table 2). Among these, ten *Blastocystis* subtypes (ST3, ST4, ST5, ST10, ST12, ST14, ST21, ST23, ST25, and ST26) were detected in herbivore species, and four subtypes (ST1, ST3, ST10, and ST21) were identified within omnivorous animals. Additionally, four subtypes, namely ST1, ST2, ST3, and ST13, were found in non-human primate species (Table 2). Notably, ST10 was the predominant one (34.27%, 49 out of 143 samples) and primarily identified in herbivorous animals. Furthermore, ST3 also emerged as a prevalent subtype, accounting for 24.48% (35 out of 143 samples) and mainly detected in omnivorous organisms, such as monkeys and black bears. Regarding subtypes ST2, ST4, ST12, ST13, ST23, and ST26, they all parasitized a single animal species and exhibited host specificity (Table 2).

### Phylogenetic analysis

**Fig. 1:**
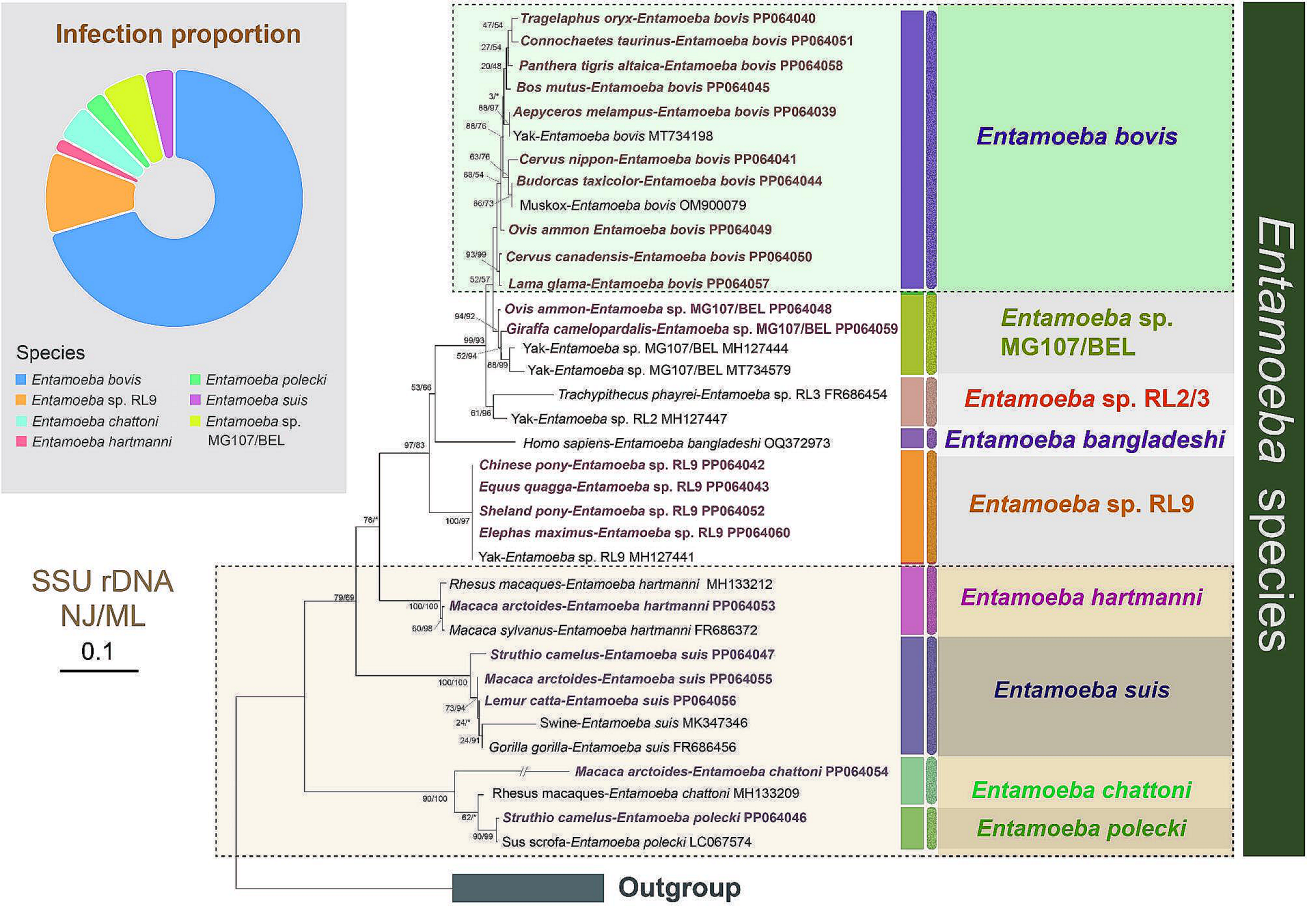
Phylogenetic trees derived from SSU rDNA data focusing on *Entamoeba* spp. identified in this study and reference strains. The numbers near nodes show the bootstrap values of NJ and ML analyses out of 1,000 replicates, respectively. The newly characterized sequences in this study are highlighted in bold brown. The scale bar corresponds to 10 substitutions per 100 nucleotide positions. The pie chart displays the *Entamoeba* species detected in this study

**Fig. 2:**
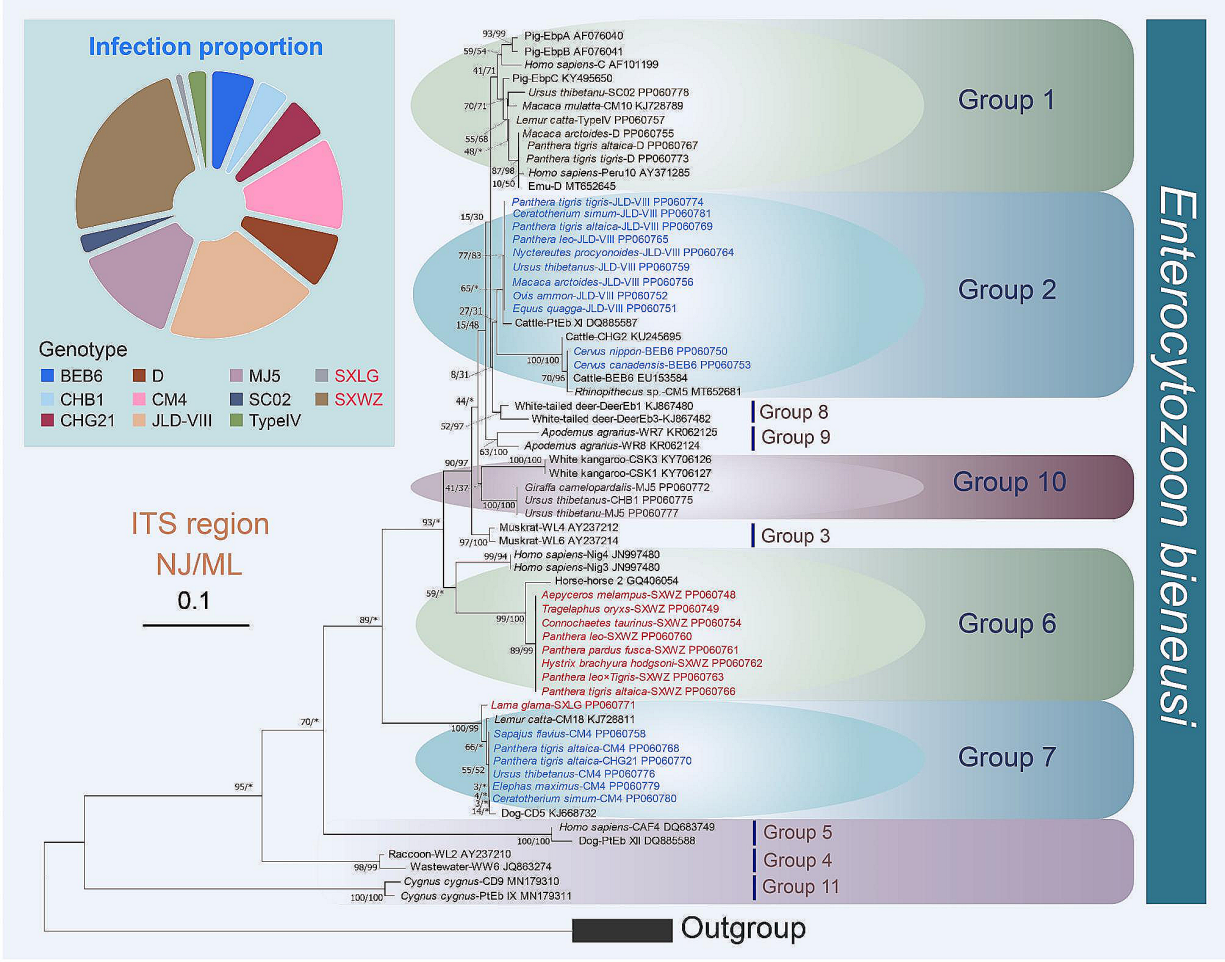
Phylogenetic trees derived from ITS data focusing on *Enterocytozoon bieneusi* identified in this study and reference genotypes. The numbers near nodes show the bootstrap values of NJ and ML analyses out of 1,000 replicates, respectively. The newly characterized sequences in this study are highlighted in brown, blue and red (novel genotypes). The scale bar corresponds to 10 substitutions per 100 nucleotide positions. The pie chart displays the *Enterocytozoon bieneusi* genotypes detected in this study

**Fig. 3:**
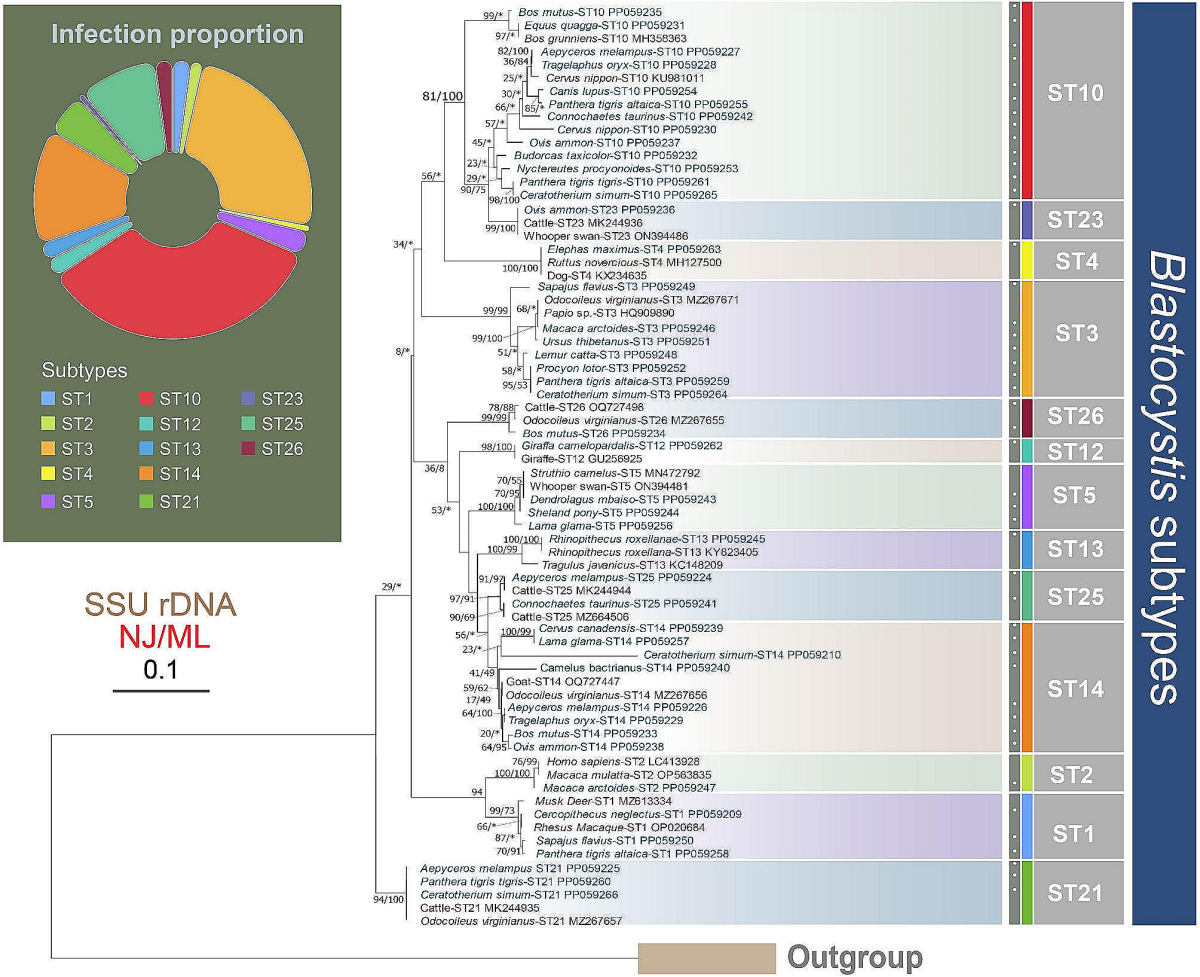
Phylogenetic trees derived from SSU rDNA data focusing on *Blastocystis* identified in this study and reference subtypes. The numbers near nodes show the bootstrap values of NJ and ML analyses out of 1,000 replicates, respectively. The newly characterized sequences in this study are highlighted in turquoise. The scale bar corresponds to 10 substitutions per 100 nucleotide positions. The pie chart displays the *Blastocystis* subtypes detected in this study

To elucidate the evolutionary relationships within these parasites, phylogenetic analyses were conducted in this study. Given that the topologies of NJ and ML trees are almost identical, only the topologies of NJ trees with bootstrap values generated from two algorithms are shown in Figs. 1, 2 and 3.

Regarding *Entamoeba* taxa, the phylogenetic tree reveals that our new sequences of seven species cluster with corresponding known sequences (Fig. 1). Except for *Entamoeba bangladeshi* and *Entamoeba chattoni*, all of the other five species form monophyletic groups, respectively. Among these taxa, *Entamoeba bovis* clusters with *Entamoeba* sp. MG107/BEL, forming a sister clade to *Entamoeba* sp. RL3 and *Entamoeba* sp. RL2. *Entamoeba chattoni* and *Entamoeba polecki* group together with each other, occupying the basal position of *Entamoeba* spp. Interestingly, all *Entamoeba* sp. RL9 sequences form parallel branches with pretty high support (NJ/ML: 100/97%), although they are derived from different hosts.

Based on ITS data, phylogenetic trees focusing on *Enterocytozoon bieneusi* were constructed, revealing 11 prominent genetic groups (Fig. 2) [21]. Among them, all genotypes SC02, Type IV, EbpA, EbpB, EbpC, Peru10, and Type C, D cluster within Group 1, the largest genotype group [21]. Subsequently, Group 1 falls outside of Group 8 and Group 2 composed of genotypes BEB6, JLD-VIII, PtEb XI, CHG2, and CM5. Genotypes MJ5 and CHB1 form parallel branches with full support and nest within Group 10. Similarly, all sequences of our new genotype SXWZ are also parallel with each other and assigned into Group 6 with high support (NJ/ML: 89/99%). For another novel genotype (SXLG) identified in this study, it clusters with genotypes CM4, CHG21, CM18, and CD5, forming the highly supported clade of Group 7 (NJ/ML:100/99%), which falls outside of the assemblage of Group 1–3 + Group 6–10 (Fig. 2).

In this study, thirteen subtypes of *Blastocystis* were detected (Fig. 3). Except for subtype 10 (ST10), all the other subtypes form monophyletic clades. Additionally, the group comprising subtypes 3, 4, 10, and 23 is sister to the assemblage of subtypes 26 + 12 + 5 + 13 + 25 + 14. Furthermore, subtypes 1 and 2 group together, falling outside of all the above subtypes, and subtype 21 is located at the basal position of *Blastocystis* (Fig. 3).

## Discussion

*Entamoeba* spp., *Enterocytozoon bieneusi*, and *Blastocystis* are prevalent enteric parasites infecting various captive wild animals, pets, and humans, leading to various diseases with symptoms of self-limiting diarrhea, dehydration, and even death in severe cases [10, 13, 20, 21, 41–43]. Previous studies have indicated that wildlife served as a crucial reservoir for the emergence and transmission of these zoonotic parasites [44].

In this study, we conducted a survey on wild animals from wildlife sanctuary near Huang Gorge of the Qinling Mountains in northwest China, to investigate the prevalence of zoonotic parasites. Our results reveal the presence of seven species of *Entamoeba*, nine genotypes of *Enterocytozoon bieneusi*, and thirteen subtypes of *Blastocystis* (Table 2), suggesting that these three parasites are widespread pathogens in wild animals at the sanctuary. In addition, the overall infection rate of parasites is 90.05% (Table 1), markedly surpassing those in most reported wildlife [36, 38, 45]. According to previous studies, the infection rate of parasites exhibited correlations with management strategy and animal density in animal protection areas [46]. In this study, we speculate that this elevated infection rate may be correlated with the expansive living environment within the wildlife sanctuary, which increases exposure opportunities and time of wildlife to the natural environment and other animals. Additionally, animal excrement may contain parasitic cysts or oocysts, contaminating water resources and posing a higher infection risk of parasites through the fecal-oral route. Moreover, animals in sanctuaries usually exhibit higher population densities compared to their natural habitats. This also increases contact between animals, the accumulation of feces containing pathogens, and the risk of parasitic transmission.

As one of the main parasites, *Entamoeba* taxa exhibit a prevalence of 54.97% (105/191) among wild captive animals in this study (Table 1). Among the seven *Entamoeba* species identified in this investigation, *Entamoeba bovis* possesses the highest prevalence rate of 70.48% (74/105) (Table 2). Notably, this species was predominantly detected in herbivorous animals, which is generally concordant with previous study, highlighting extensive distribution in wild cervids [47]. *Entamoeba* sp. MG107/BEL and *Entamoeba* sp. RL9 were found to infect two and four species of feral animals, respectively (Table 2). These species have been previously reported in non-human primates and ruminants such as yaks [48, 49]. In this study, except for ruminant animals (giraffes), non-ruminant animals such as zebras and elephants were also found to be infected by *Entamoeba* sp. MG107/BEL and *Entamoeba* sp. RL9, which indicates that these two species could infect more animal hosts, expanding our knowledge on their reservoir diversity. Additionally, *Entamoeba hartmanni* and *Entamoeba chattoni*, known to infect children and non-human primates [40, 48, 50], were also detected in macaques in this study (Table 2). The remaining two *Entamoeba* isolates, namely *Entamoeba polecki* and *Entamoeba suis*, were traditionally associated with pigs according to previous studies [51, 52]. However, our study reveals their presence in primates and ostriches (Table 2), suggesting that these two *Entamoeba* species may have a more diverse range of animal hosts.

Regarding the parasite *Enterocytozoon bieneusi*, it exhibits the prevalence of 35.08% (Table 1), with nine known genotypes of *Enterocytozoon bieneusi* and two novel genotypes (SXWZ and SXLG) identified in present study. These novel genotypes are phylogenetically categorized into Group 6 and Group 7, respectively (Fig. 2). Previous research has indicated that Group 6 and Group 7 tend to be more host-specific, given that most genotypes of them were only found in their originally reported host animals [21]. In concordance with this, the novel genotype SXLG was exclusively identified in alpacas (Table 2) in this work. On the other hand, the novel genotype SXWZ, detected in multiple artiodactyl and carnivorous animals such as tigers, lions, leopards, sheep, and horses, suggests a broader host adaptability of this genotype and complex interactions between host animals and the parasite. However, additional investigations are required to comprehensively understand the infection characteristics of this novel genotype. In addition to the novel genotypes, this study has also identified several known genotypes of *Enterocytozoon bieneusi*. Genotypes D, SC02, and Type IV were detected in black bears, monkeys, and tigers, respectively (Table 2). According to phylogenetic analyses (Fig. 2), these three genotypes fall into Group 1, well-known for its association with human infections [21]. This suggests a potential risk of transmission to humans and the likelihood of infections spreading between different wildlife species. Genotypes JLD-III and BEB6, categorized into Group 2 (Fig. 2), were initially believed to infect only ruminant species [53]. However, the presence of genotype BEB6 in deer (Table 2) aligns with previous findings where it was identified in wild deer and humans [54, 55]. This implies that deer might serve as a potential reservoir for genotype BEB6, posing a risk of transmission between wild animals and humans.

*Blastocystis*, a parasitic protozoan found in the gastrointestinal tracts of animals and humans, is well-known for its prevalence and diverse subtypes [42]. The investigation here reveals an infection rate of 74.87% for *Blastocystis*, with a total of thirteen subtypes (ST1–5, ST10, ST12–14, ST21, ST23, ST25–26) (Tables 1 and 2). Among these *Blastocystis* subtypes, ST1–10, ST12, ST14, ST16, and ST24 are known to have the ability to infect humans, particularly ST1–4 predominantly infecting humans (> 90%) [28, 32, 56]. Previous research has demonstrated that these subtypes (e.g., ST1, ST2, ST3, and ST5) were not only detected in animals but also in their in-contact humans [35, 57]. Although fecal samples of animal caretaker are not included in this study, over 50% of the detected genotypes (ST1–5, ST10, ST12, ST14) exhibit zoonotic characteristics, suggesting significant possibilities of parasite transmission between animals and humans (Table 2) [5, 56]. The host of remaining subtypes (ST13, ST21, ST23, ST25–26) detected in this study are concordant to those reported in previous studies (Table 2) [29, 32, 58–60].

The data given in this study indicates the presence of three zoonotic parasites among wild captive species in the wildlife sanctuary in northwest China with high biodiversity and extensive, close-to-nature habitats. Within the sanctuary comprising multiple grazing areas, infected animals may excrete parasites through feces into the external environment, contaminating water sources or food. Subsequently, other animals may become infected through the fecal-oral route, thereby perpetuating a cycle of infection within the sanctuary. At the same time, individuals who frequently visit or take care of animals usually engage in direct interactions with wildlife, which may contribute to an increased risk of parasite transmission between animals and humans. This highlights the need of measures to protect both animals and humans from infections derived from consumption of contaminated food or water containing pathogens. Certainly, the findings underscore the importance of additional research to further investigate parasite infections and transmission pathways among wildlife, caretakers, and visitors. This would be helpful to understand the transmission mechanisms and develop effective prevention strategies against zoonotic parasites across different species. Therefore, such researches can contribute to promoting the overall well-being of both humans and animals in wildlife sanctuary, parks and similar settings.

## Conclusions

In this study, we investigated the prevalence and genetic diversity of three zoonotic parasites, *Entamoeba* spp., *Enterocytozoon bieneusi*, and *Blastocystis*, in captive wild animals from a sanctuary in Shaanxi Province, northwest China. The identification and distribution patterns of zoonotic *Enterocytozoon bieneusi* and *Blastocystis* suggest the potential of transmission between animals and humans, indicating a potential outbreak risk of zoonotic diseases originating from these wild animal reservoirs.

## Methods

### Sample collection

**Fig. 4:**
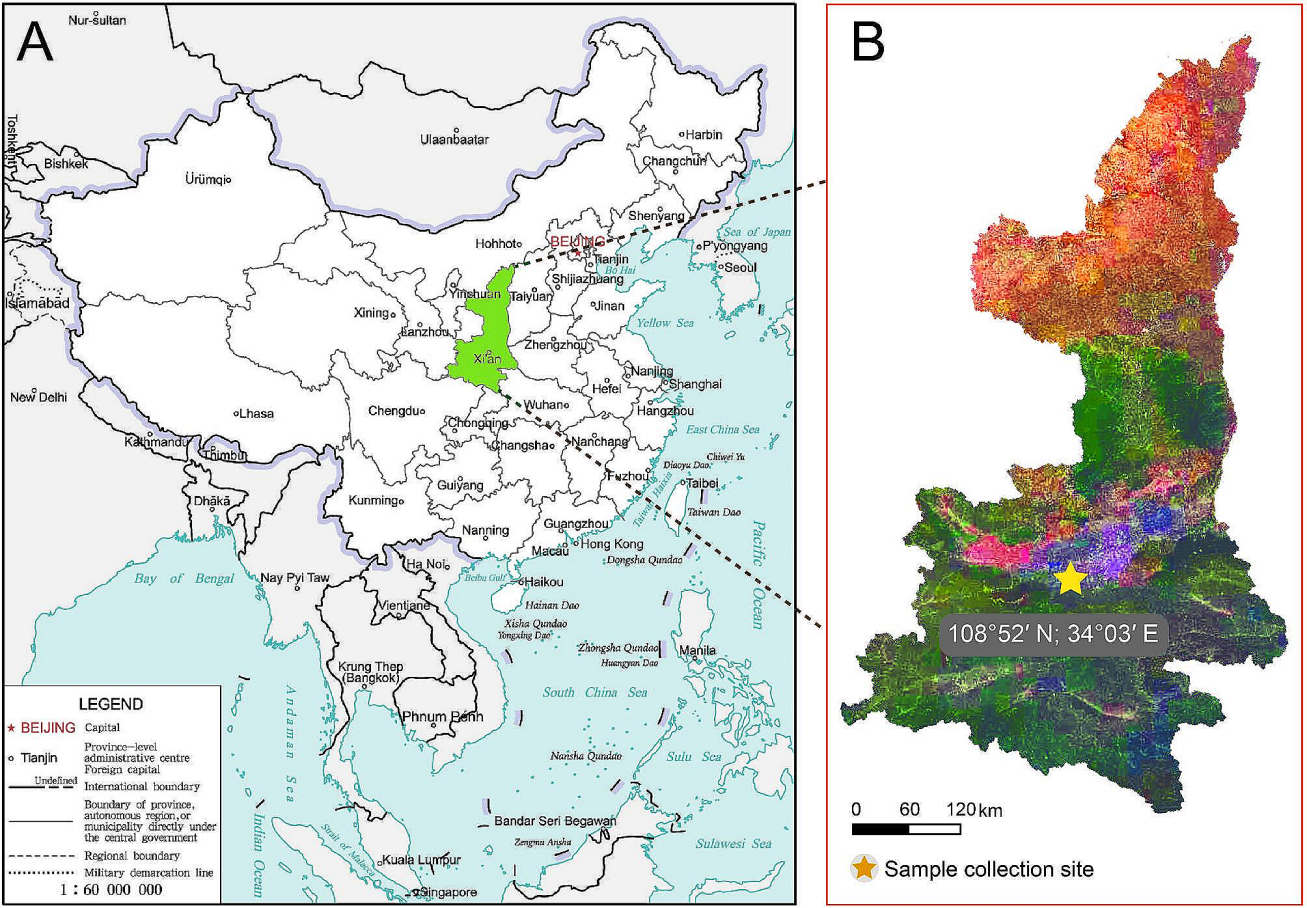
Location of the sampling site in this study. **A**, map to show the collecting sites in China. The map of China [drawing review number: GS (2019)1652] cited from the MAP WORD (www.tianditu.gov.cn). **B**, map to show the collecting sites in Shaanxi province, China

During the period from February 2023 to May 2023, a total of 191 fecal samples were collected from 37 captive wild animals (see Additional file 1) at the wildlife sanctuary (108°52′N, 34°03′E) located near the Huang Gorge of the Qinling Mountains in Shaanxi province, northwest China (Fig. 4). Immediately following animal defecation, fresh fecal samples, approximately 30–50 g, were carefully collected without contacting the ground. In addition, we collected samples based on different enclosures and habitats, with efforts made to avoid collecting fecal samples from the same animal more than once whenever possible. Subsequently, all fecal specimens were preserved in 2.5% potassium dichromate solution at 4 °C until further processing.

### DNA extraction

Each fecal specimen was washed three times with distilled water, followed by centrifugation at 3,500×g for 5 min to remove the potassium dichromate. Subsequently, approximately 200 mg of the sample was thoroughly homogenized with 200 mg of glass beads by vortex. DNA extraction was carried out utilizing the E.Z.N.A. Stool DNA Kit (Omega Biotek Inc., Norcross, GC), following the manufacturer’s instructions.

### PCR amplification and sequencing

PCR amplifications were conducted to obtain the internal transcribed spacer (ITS) region and small subunit ribosome RNA (SSU rRNA) gene for the identification of *Enterocytozoon bieneusi* and *Blastocystis* + *Entamoeba* spp., respectively [61–64]. Details of the primers and annealing temperatures used in this study are present in Table S2 (Additional file 2). The KOD Plus DNA polymerase (Toyobo Co., Ltd., Osaka, Japan) was employed, and each PCR amplification included a positive control (known DNA of *Entamoeba bovis*, *Enterocytozoon bieneusi* genotype JLD-VIII and *Blastocystis* subtype 10) and a negative control (no DNA). To ensure precision, amplifications were conducted with three independent reactions for each sample. All positive PCR products were bidirectional sequenced at Sangon Biotech (Shanghai) Co., Ltd. Subsequently, gene sequences were assembled using SeqMan ver. 7.1.0 [65, 66], and then aligned by BioEdit ver. 7.0.9.0 [67, 68] and BLAST (https://blast.ncbi.nlm.nih.gov/Blast.cgi) to verify accuracy.

### Phylogenetic analysis

Our newly obtained sequences were integrated with those of relevant species, genotypes, or subtypes, creating three databases for phylogenetic analyses. Subsequently, each dataset was aligned and edited using BioEdit ver. 7.0.9.0 [67, 68], resulting in alignments with lengths of 650 bp (*Entamoeba* spp.), 617 bp (*Blastocystis*), and 414 bp (*Enterocytozoon bieneusi*), respectively. The neighbor-joining (NJ) tree was constructed using MEGA-X software [69, 70] with the Kimura two-parameter model, and Maximum Likelihood (ML) analysis was performed using IQ-TREE ver. 2.2.2.7 [71, 72] with the models TN + F + I + R2 (*Entamoeba* spp.), HKY + F + G4 (*Enterocytozoon bieneusi*), and TPM3u + F + R5 (*Blastocystis*), respectively. To assess the reliability of the trees, 1,000 bootstrap replicates were conducted for both NJ and ML trees. All new sequences generated in this study have been deposited in the GenBank database, and the accession numbers are displayed on the phylogenetic trees (Figs. 1, 2 and 3). The SSU rRNA gene of *Rhizomastix bicoronata* (KP343638), *Histomonas meleagridis* (AJ920323), and ITS gene of *Enterocytozoon bieneusi* genotype CSK2 (KY706128) were employed as outgroup for the phylogenetic trees of *Entamoeba* spp., *Blastocystis*, and *Enterocytozoon bieneusi*, respectively.

### Statistical analysis

To illustrate the prevalence of parasites, we utilized SPSS ver. 26.0 (SPSS Inc., Chicago, IL, USA) for computation of frequency and percentage of parasitic infection, as well as the 95% confidence intervals (CIs) of infection rates. Statistically significant differences were identified when *p*-values were less than 0.05.

### Electronic supplementary material

Below is the link to the electronic supplementary material.

Supplementary Material 1

Supplementary Material 2

## Data Availability

Nucleotide sequences were deposited in GenBank under the following accession numbers: PP064039-PP064060, PP060748-PP060781, PP059224-PP059266 and PP059209-PP059210.

## Abbreviations

SSU rRNA: Small subunit rRNA
CI: Confidence intervals
NJ: Neighbor-Joining
ML: Maximum likelihood
ITS: Internal transcribed spacer
SNPs: Single nucleotide polymorphisms
