# Supplementary material for: Molecular characterization and zoonotic potential of Entamoeba spp., Enterocytozoon bieneusi and Blastocystis from captive wild animals in northwest China
Source: BMC Vet Res. 2024 Jul 10;20:309. doi: 10.1186/s12917-024-04172-y (PMC11234763; doi:10.1186/s12917-024-04172-y)
Supplement: Supplementary file 2 — Supplementary Material 2 [file 12917_2024_4172_MOESM2_ESM.doc]

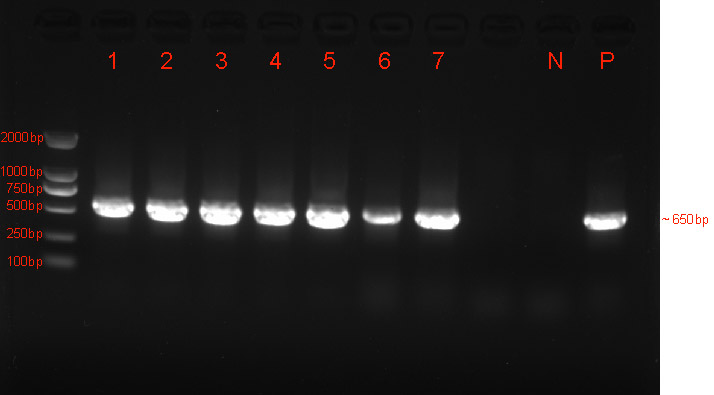


Figure S1 Agarose gel electrophoresis of PCR products amplified at the SSU rRNA locus of *Entamoeba*. 1-7: samples. N: negative control. P: positive control.


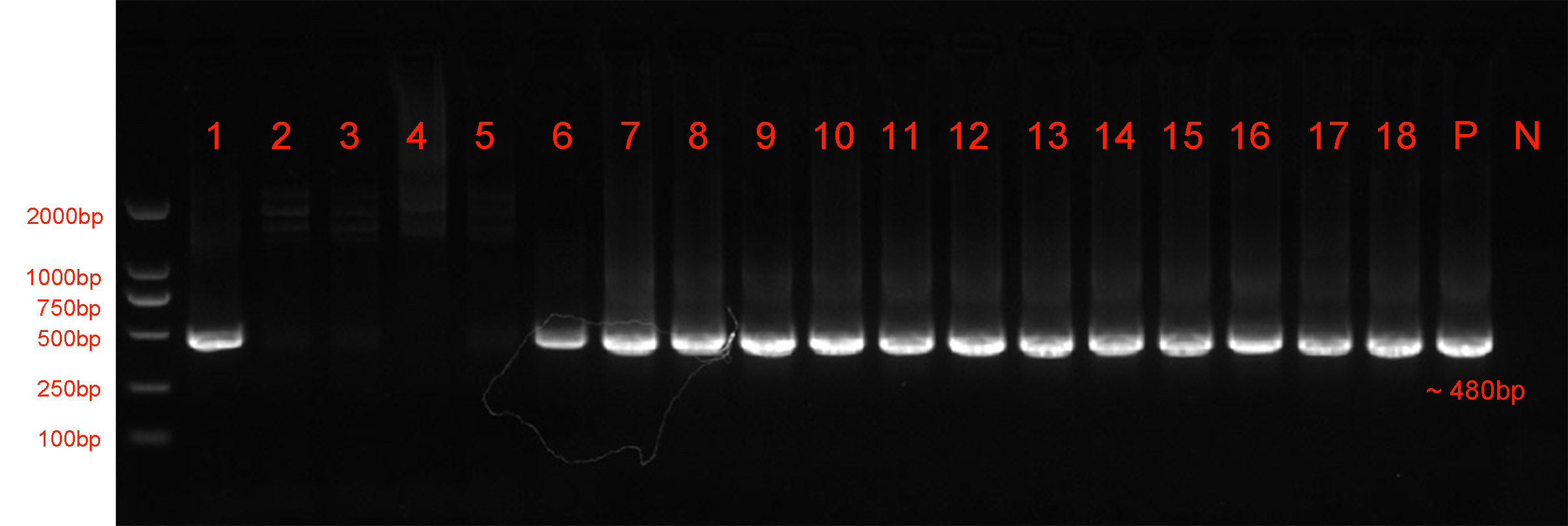


Figure S2 Agarose gel electrophoresis of PCR products amplified at the SSU rRNA locus of *Blastocystis*. 1-18: samples. N: negative control. P: positive control.


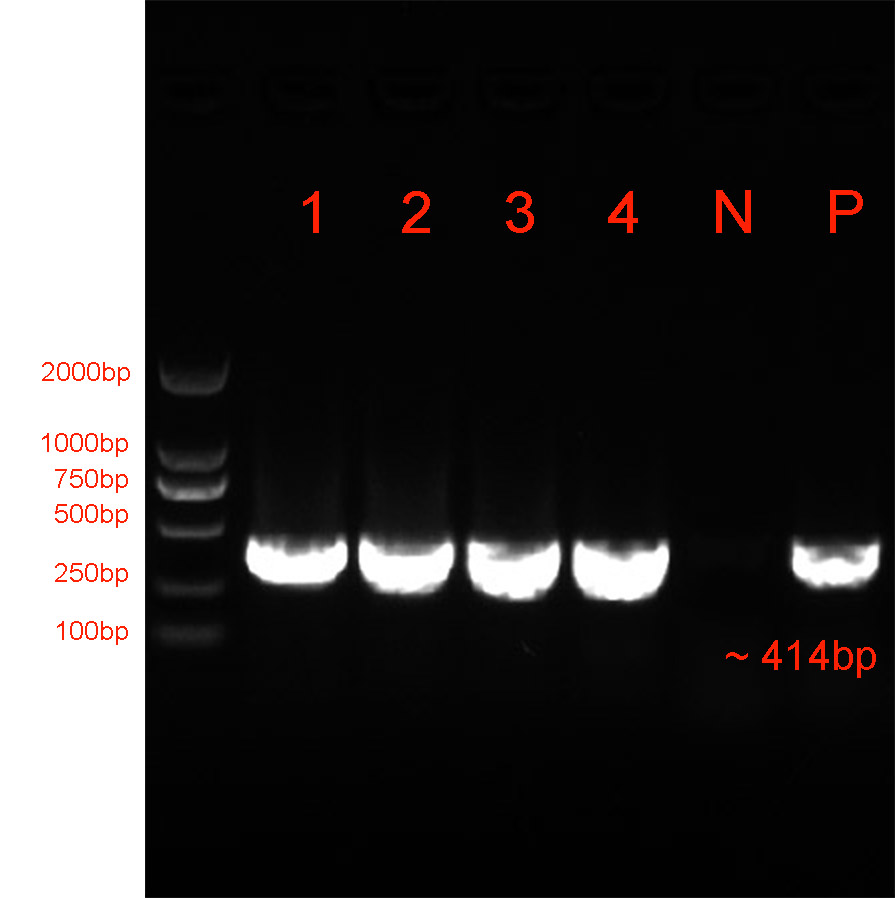


Figure S3 Agarose gel electrophoresis of PCR products amplified at the ITS locus of *Enterocytozoon bieneusi*. 1-4: samples. N: negative control. P: positive control.


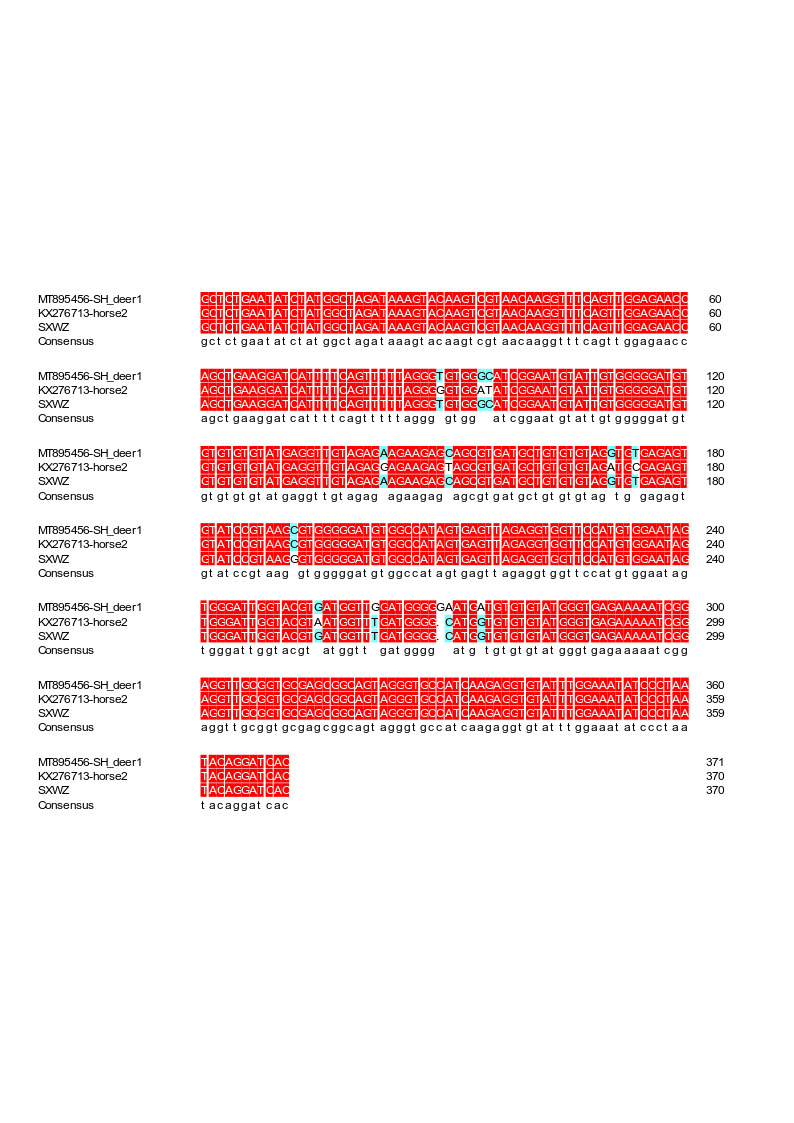


Figure S4 Sequences alignment between new genotype SXWZ and other genotypes of *Enterocytozoon bieneusi*.


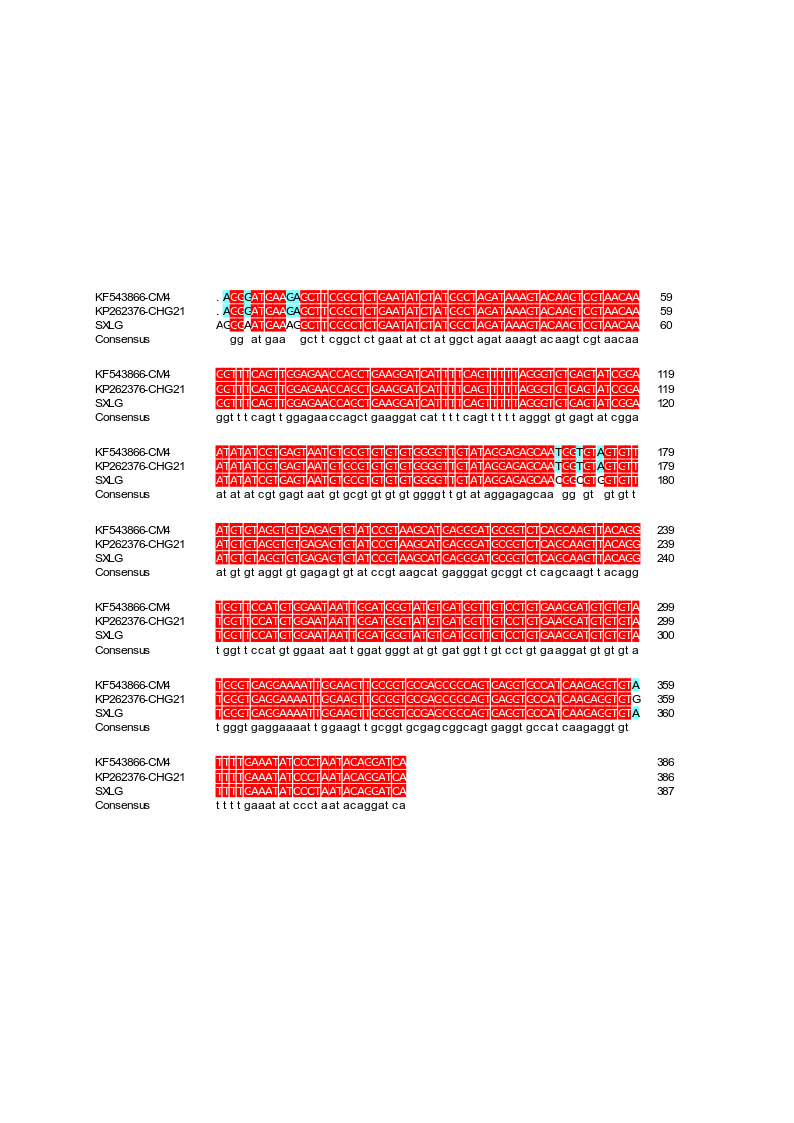


Figure S5 Sequences alignment between new genotype SXLG and other genotypes of *Enterocytozoon bieneusi*.

**Table S1** Primers for PCR amplification used in this study

| **Species** | **Primers** | **Sequences (5′- 3′)** | **Annealing temperature (ºC)** | **References** |
| --- | --- | --- | --- | --- |
| *Enterocytozoon bieneusi* | EBITS3 | GATGGTCATAGGGATGAAGAGCTT | 57 | [47] |
|  | EBITS4 | TATGCTTAAGTCCAGGGAG |  |  |
|  | EBITS1 | AGGGATGAAGAGCTTCGGCTCTG | 55 |  |
|  | EBITS2.4 | AGTGATCCTGTATTAGGGATATT |  |  |
| *Blastocystis* | RD5 | GGAAGCTTATCTGGTTGATCCTGCCAGTA | 55 | [44] |
|  | RD3 | GGGATCCTGATCCTTCCGCAGGTTCACCTAC |  |  |
|  | Bla1 | GGAGGTAGTGACAATAAATC | 54 | [45] |
|  | Bla2 | TGCTTTCGCACTTGTTCATC |  |  |
| *Entamoeba* spp. | Entam1 | GTTGATCCTGCCAGTATTATATG | 57 | [48] |
|  | Entam2 | CACTATTGGAGCTGGAATTAC |  |  |
